# Preconditioning Methods to Improve Mesenchymal Stromal Cell-Derived Extracellular Vesicles in Bone Regeneration—A Systematic Review

**DOI:** 10.3390/biology11050733

**Published:** 2022-05-11

**Authors:** Fernanda Campos Hertel, Aline Silvestrini da Silva, Adriano de Paula Sabino, Fabrício Luciani Valente, Emily Correna Carlo Reis

**Affiliations:** 1 Veterinary Department, Federal University of Viçosa, Vicosa 36570-900, Brazil; fernanda.hertel@ufv.br (F.C.H.); aline.silvestrini@ufv.br (A.S.d.S.); fabriciovalente@ufv.br (F.L.V.); 2 Department of Clinical and Toxicological Analysis, Federal University of Minas Gerais, Belo Horizonte 31270-901, Brazil; adriansabin@ufmg.br

**Keywords:** cell communication, conditioned medium, exosomes, microenvironment, osteogenesis

## Abstract

**Simple Summary:**

The evidence of the therapeutic effects of mesenchymal stromal cells (MSCs), so-called stem cells, in several diseases relies mostly on the substances they secrete, including their extracellular vesicles (EVs). EVs are an important component of cell communication and they carry a cargo that is similar to their parent cell. Cells respond differently based on their microenvironment, and so it is expected that the therapeutic potential of these vesicles can be modulated by the enrichment of their parent cell microenvironment. With this in mind, we conducted a systematic search for papers that preconditioned MSCs and collected their EVs to assess their potential to favor bone formation. The results showed different methods for MSC preconditioning, including chemical induction, culture conditions, and genetic modifications. All methods were able to improve the therapeutic effects of the derived EVs for bone formation. However, the heterogeneity among studies—regarding the type of cell, EV concentration, and scaffolds—made it difficult to compare fairly the types of preconditioning methods. In summary, the microenvironment greatly influences MSCs, and using preconditioning methods can potentially improve the therapeutic effects of their derived EVs in bone regeneration and other bone diseases.

**Abstract:**

Mesenchymal stromal cells (MSCs) have long been used in research for bone regeneration, with evidence of their beneficial properties. In the segmental area of MSC-based therapies, MSC-derived extracellular vesicles (EVs) have also shown great therapeutic effects in several diseases, including bone healing. This study aimed to assess whether the conditioning of MSCs improves the therapeutic effects of their derived extracellular vesicles for bone regeneration. Electronic research was performed until February 2021 to recover the studies in the following databases: PubMed, Scopus, and Web of Science. The studies were screened based on the inclusion criteria. Relevant information was extracted, including in vitro and in vivo experiments, and the animal studies were evaluated for risk of bias by the SYRCLE tool. A total of 463 studies were retrieved, and 18 studies met the inclusion criteria (10 studies for their in vitro analysis, and 8 studies for their in vitro and in vivo analysis). The conditioning methods reported included: osteogenic medium; dimethyloxalylglycine; dexamethasone; strontium-substituted calcium silicate; hypoxia; 3D mechanical microenvironment; and the overexpression of miR-375, bone morphogenetic protein-2, and mutant hypoxia-inducible factor-1α. The conditioning methods of MSCs in the reported studies generate exosomes able to significantly promote bone regeneration. However, heterogeneity regarding cell source, conditioning method, EV isolation and concentration, and defect model was observed among the studies. The different conditioning methods reported in this review do improve the therapeutic effects of MSC-derived EVs for bone regeneration, but they still need to be addressed in larger animal models for further clinical application.

## 1. Introduction

Bone repair is a complex and multistep process that follows a defined temporal and spatial sequence [1]. Under the ideal conditions, bone can regenerate and return to its original state [2]. However, a disruption in this process, often by extensive tissue damage, disease, advanced age, developmental deformity, tumor resection, or infection, can lead to undesirable bone-healing effects [1], such as delayed union and non-union. Currently, the methods for treating these conditions rely on bone grafting (autologous or allogeneic) and distraction osteogenesis; however, there are still complications related to these techniques [3].

The rise of mesenchymal stem/stromal cells in the last five decades has brought great interest to their regenerative potential. First, it was believed that MSCs could function as a replacement for the host tissue, and it is now recognized that the functions of these cells are mostly related to their secreted immunomodulatory and bioactive factors in response to the local microenvironment in which they are implanted [4,5,6], with evidence of their therapeutic effects in bone regeneration [7,8,9].

Despite the promising results regarding MSC research, this field still faces difficulties in translating its use for clinical applications, including determining the specific phenotype for each disease, autologous vs. allogeneic cells, cell dose, the frequency of application, delivery route, the ideal microenvironment for cells to exert their effects, and cryopreservation methods [10]. Along with the fact that MSCs act throughout paracrine mechanisms and in an attempt to overcome these problems, cell-free therapies such as MSC-derived extracellular vesicles have become strongly established in the landscape of regenerative medicine.

Extracellular vesicles (EVs) is the generic name for nano-sized vesicles that are naturally released from cells and can be divided into two types: exosomes and microvesicles. Based on their biogenesis, exosomes originate from the endosomal system, and microvesicles are shed from the plasma membrane [11,12]. These nanoparticles are known to participate in cell communication, by transferring signaling information as proteins, lipids, RNA, and DNA, and these contents can impact the functional property of the target cell [13,14].

The application of MSC-based therapies in bone regeneration has been demonstrated, with promising results and challenges to overcome, such as determining the ideal cell type, culture conditions, dosage, immunocompatibility (reviewed in [15]). Among MSC-based therapies, research on MSC-derived EVs emerged as a promising cell-free approach. Several studies have been conducted in the field of bone regeneration, including fracture healing [16,17], calvarial critical-sized defects [18,19,20,21], osteoporosis [22], and the necrosis of the femoral head [23].

Unraveling the microenvironment changes in diseased tissues is fundamental for applications of cell-based therapies, as this environment can dictate the fate of the applied therapy. Researchers have been trying to enhance the therapeutic potential of MSCs by conditioning these cells towards the modulation of target diseases [24], mimicking their activation upon encountering a healing microenvironment. This can be achieved by biophysical cues (three-dimensional cultures, mechanical tension, electric pulsing); biochemical cues (hypoxia, cytokines, growth factors); and cellular reprogramming (protein or microRNA overexpression) [25]. Therefore, this study aimed to systematically review the available literature to answer the following question: does the conditioning of MSCs improve the therapeutic effects of MSC-derived EVs for bone regeneration?

Due to the pathological differences among the several bone-related diseases (such as osteoporosis, osteogenesis imperfecta, femoral head necrosis, or periodontal-related disease), only the studies reporting methods to enhance bone regeneration in the bone defect spectrum were included. This allowed us to draw a more straightforward comparison between conditioning methods.

## 2. Materials and Methods

This systematic review was conducted following the key principles recommended in the PRISMA statement [26]. The PICO format was used to formulate the research question, where the population (P) was that of in vitro or in vivo studies with a focus on osteogenesis/angiogenesis and bone regeneration, the intervention (I) was the treatment with EVs derived from preconditioned MSCs, the comparison (C) was the animals or cells treated with control EVs (derived from unconditioned MSCs), and the outcome (O) was osteogenesis/angiogenesis for in vitro studies and bone regeneration for in vivo studies.

### 2.1. Search Strategy

Articles were selected from PUBMED, SCOPUS, and Web of Science until February 2021. The search was conducted using the following terms: (exosomes OR “extracellular vesicles” OR EVs OR microvesicles) AND (osteogenesis OR “bone regeneration” OR fracture OR “bone healing” OR “bone repair”) AND (“mesenchymal stem cells” OR “mesenchymal stromal cells” OR “stem cells” OR MSCs). The specific search strategies for each database can be found in Appendix A.

### 2.2. Eligibility Criteria

The articles screened were included in this study based on the following criteria: articles that reported the treatment with preconditioned MSC-derived EVs in vitro and/or in vivo; articles that reported the assessment of osteogenesis in vitro and/or in vivo. Applied exclusion criteria were: articles that reported the use of EVs from other cells sources; articles that reported the use of an animal model with induced disease (such as osteoporosis, obesity, femoral head necrosis, periodontal-related diseases); articles that did not use EVs as treatment; treatment associating MSCs with EVs; articles that did not report a control group of EVs; articles that did not report conditioning of MSCs; review articles; conference abstracts; protocols.

### 2.3. Study Selection

All retrieved records were first uploaded into a support tool for systematic review (StArt—State of the Art through Systematic Review) followed by removal of duplicate records. All articles were screened for eligibility by two investigators. First, the articles were screened by title and abstract; then, the full-text articles that met the inclusion criteria were recovered and screened for inclusion.

### 2.4. Data Extraction

In this systematic review, both in vitro and in vivo data were extracted, when available. The general information extracted from the articles was: author, title, year of publication, journal, and country. Technical information extracted from in vitro analysis was: source of EVs; type of EV; isolation and characterization methods; EV markers; size distribution; conditioning method; control EVs; type of cell treated; analysis reported, treatment groups; EV concentration; treatment duration; and outcomes. Secondary outcomes for in vitro results were also extracted (miRNA/circRNA profile, signaling pathway analysis). For in vivo analysis, the extracted data were: type of animal model; bone defect model; treatment groups; treatment used (exosome concentration, vehicle, scaffold, treatment duration); analysis reported; and outcomes.

### 2.5. Risk of Bias Assessment

The risk of bias for animal studies was assessed using the Systematic Review Centre for Laboratory animal Experimentation (SYRCLE) tool [27].

## 3. Results

### 3.1. Search Results

**Figure 1:**
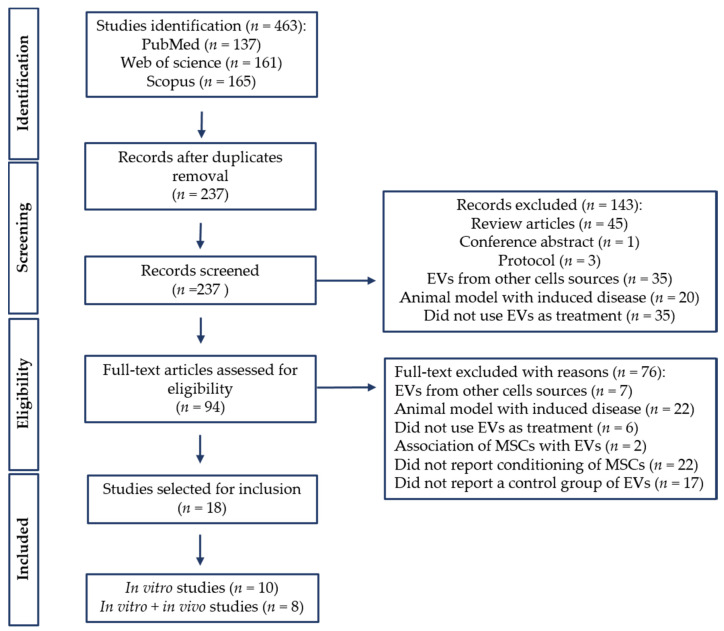
PRISMA flow diagram of literature search and selection process. PRISMA, Preferred Reporting Items for Systematic Reviews and Meta-Analyses.

A total of 463 studies were retrieved in the initial database search. After duplicate removal, 237 studies were screened by title and abstract, resulting in the removal of 143 studies. After full-text examination, a total of 18 studies met the inclusion criteria and were included in this systematic review (Figure 1). Among these studies, three articles that reported in vitro and in vivo experiments did not meet the inclusion criteria for the in vivo experiments [28,29,30]; however, the in vitro analysis was included in this review. For this reason, 10 studies were extracted for in vitro analysis [28,29,30,31,32,33,34,35,36,37], and 8 studies were extracted for in vitro and in vivo analysis [17,18,20,21,38,39,40,41].

### 3.2. Study Characteristics

The studies included were published from 2016 to 2021; the year 2020 was the year with the most publications, indicating that conditioning MSCs before EV collection is becoming a more common approach. Several journals published these studies, from general science to specific regenerative medicine journals, and China was the country with the highest number of published studies. Detailed information can be found in the supplementary material (Table S1).

### 3.3. Conditioning Methods

The conditioning methods reported varied among studies (Table 1). The induction of MSCs with an osteogenic medium was reported by nine studies [29,31,32,33,34,35,36,37,40], four of which associated it with serum deprivation before EV collection [32,34,35,37]. The other methods included other chemicals, such as dimethyloxalylglycine [20], dexamethasone [30], and strontium-substituted calcium silicate [41]; culture conditions such as hypoxia [17] and a 3D mechanical microenvironment [39]; genetic modifications such as miR-375-overexpressing ACSs [38]; and, finally, genetic modifications associated with serum deprivation such as BMP2-overexpressing BMSCs [21] and mutant HIF-1α-modified BMSCs [18,28].

### 3.4. EV Isolation and Characterization

A summary of the EV isolation and characterization methods reported by the included studies can be found in Table 2.

Human cells were the predominant source for EV isolation, present in 14 of the 18 studies. Three studies used EVs derived from rats, and only one study used EVs derived from rabbit cells. The origin of these cells was varied: human bone marrow mesenchymal stem cells (BMSCs) [20,21,32,35,37]; human adipose-derived stem cells (ASCs) [29,34,38]; human periodontal ligament stem cells (PDLSCs) [33,39]; human umbilical cord mesenchymal stem cells (UCMSCs) [17]; human placental stem cells (PSCs) [32]; human mesenchymal stem cells (MSCs, no origin reported) [36,40]; rat BMSCs [18,30,41]; and rabbit BMSCs [28].

Based on the type of EV, 83% of the studies (*n* = 15) characterized these vesicles as exosomes [17,18,20,28,29,31,33,34,35,36,37,38,39,40,41], followed by two studies that referred to the isolated vesicles by the generic name “extracellular vesicles” [21,32] and one study that reported the use of microvesicles [30].

Ultracentrifugation was the isolation method most frequently reported (44.4%, *n* = 8) [20,29,31,33,34,35,36,38]. An isolation kit was also used in many studies (27.8%, *n* = 5) [21,28,37,39,40], followed by the combination of ultracentrifugation and ultrafiltration (22.2%, *n* = 4) [17,18,32,41] and differential centrifugation (5.6%, *n* = 1) [30]. To properly characterize the extracellular vesicles, most articles performed transmission electron microscopy, nanoparticle tracking analysis, and Western blotting to assess morphology, size distribution, and EV-associated markers, respectively. Other methods with the same purpose were used in some studies, such as flow cytometry [30,31], dynamic light scattering [32], tunable resistive pulse sensing [18], and atomic force microscopy [40]. Four articles did not report EV-associated markers [32,35,36,37], and one article performed only TEM [37]. The most commonly reported EV-associated markers were CD9, CD63, CD81, and TSG101. Regarding the morphology and size of the EVs, most studies reported round or cup-shaped EVs with a size range of 30–200 nm, and the studies referred to them as “exosomes”; two articles reported EVs with a size > 200 nm and reported “extracellular-vesicles” < 262 nm [32] and “microvesicles” < 400 nm [30]. It is worth noting that the process of isolating EVs was very heterogeneous among the studies, with different centrifugation cycles and speeds or the use of different types of exosome-free serum (home-made or commercially acquired).

### 3.5. In Vitro Studies

All studies included in this review reported some experiments in vitro (Table 3). The most frequent cell type used as the subject of treatment were human BMSCs [21,29,32,35,37,38,39], followed by HUVECs [17,20,28,41], hMSCs [36,40], rat BMSCs [18,33], rabbit BMSCs [28], human ASCs [34], DPSCs [31], MC3T3 [30], hFOB 1.19 [17], and RAW 264.7 [35]. The type of cell was chosen according to the focus of the conditioning method, e.g., hypoxic MSC-derived exosomes were used to treat HUVECs and analyze the potential for angiogenesis, and osteogenically conditioned exosomes were used in the osteogenic differentiation of BMSCs, ASCs, or PDSCs.

The presence of a group with unconditioned EVs (unconditioned compared to the principal method in the study) was mandatory in the studies included in this review, so that a fair comparative analysis would be possible. Generally, the control EV groups in the studies were isolated from untreated cells [17,20,29,30,33,36,38,39,40,41]. However, in some studies, when serum deprivation was applied, the control EV group was isolated from cells with serum deprivation, varying from 12 to 72 h of additional culturing [21,28,32,34,35,37]. Only one study reported the conditioning of MSCs with osteogenic medium without serum deprivation, but the EV control was collected from the serum-free medium [31], and one study did not report the culture method of the control EVs [18].

In the majority of the studies, osteogenic differentiation was detected by alizarin red staining [18,28,30,31,32,33,34,36,38,39,40]. Another common analysis was ALP activity and osteogenic marker expression. To evaluate angiogenesis, cell proliferation, migration, and tube formation assays were performed in all studies that used HUVECs [17,20,28,41]. Only one study evaluated the immunoregulatory role of exosomes and did so by analyzing the expression of cytokines in RAW 264.7 cells [35]. It is important to note that some studies focused on the evaluation of angiogenesis in vitro, not in osteogenesis (Liang et al., 2019; Liu et al., 2021; W. Liu et al., 2020). However, this was complementary to the in vivo experiments on bone defects.

To analyze osteogenic differentiation, cells were cultured in proliferation medium (PM) or osteogenic medium (OM) and treated with EVs. Only three studies analyzed the osteogenic induction of EVs both in OM and PM [29,33,38]; five studies performed the osteogenic induction of EVs only in PM [21,30,31,36,40]; and five studies analyzed the effects of EVs in OM only [18,32,34,35,39]. One study did not report the type of medium in which the cells were cultured during the treatment [37].

Regarding the treatments in the in vitro analysis, the concentration of EVs ranged from 10 to 200 μg/mL. The duration of the treatments varied between studies; however, ALP activity was analyzed from 7 to 14 days of culture; ARS up to 21 days of culture; and osteogenic markers generally from 3 to 21 days of culture.

### 3.6. In Vivo Studies

A total of eight studies with in vivo experiments met the inclusion criteria for this review (Table 4). Seven of them reported the use of rats as animal models, of which six studies used Sprague–Dawley rats [18,20,38,39,40,41] and one study did not report the strain [21]. Only one study used mice as the animal model, though it also did not report the strain [17]. Regarding the defect model, four studies performed bilateral calvaria defects [18,20,21,38]; one study performed a femoral fracture model in mice [17]; one study performed an alveolar bone defect [39]; one study performed a segmental radius defect [40]; and one study performed a distal femur defect [41]. Notably, the animal models did not vary in size, and the defect models among the studies did not explore critical-sized defects in long bones or larger animals.

The treatment groups were homogeneous among studies regarding the presence of a conditioned exosome-treated group, an unconditioned exosome-treated group, and a control group (generally with the scaffold alone, except for [17], which used PBS as a carrier). One study reported a blank defect in all treated animals [38], one study reported a positive control group treated with recombinant human BMP2, and one study reported a group treated with hMSCs seeded in a titanium scaffold [40].

The concentration of EVs applied to the defects varied greatly between studies. The studies that reported calvaria defects ranged from 1 to 200 μg of exosomes. The lowest concentration was reported by [38], applying 20 μL at 50 μg/mL; Liang et al. and Ying et al. [18,20] reported 100 μg/200 μL and 200 μg of exosomes, respectively; and, differently from other studies, Huang et al. [21] reported a concentration of EV particles of 5 × 108 EVs/50 μL. One study did not report the EV concentration applied to the defects [40]. The EV concentration in other studies with different defect models ranged from 100 μg/μL [39,41] to 200 μg/μL [17].

All studies reported different carriers for EV application, including: PBS [17], ceramic-based scaffolds (HA, β-TCP) [18,20], collagen type I [21], protein-based scaffold (silk fibroin, Matrigel™) [39,41], hydrogel [38], and titanium-based scaffold [40]. Treatment duration varied from 7 days to 12 weeks. Liu et al. [17] reported a femur fracture model in mice with only a 7-day duration of treatment. The time points for different analyses were also reported; Huang et al. [21] reported a 3-time-point analysis (4, 8, and 12 weeks), and Yu et al. [39] and Zhai et al. [40] reported a 2-time-point analysis (3/6 weeks and 4/12 weeks, respectively).

Imaging analysis of bone regeneration was performed by all studies with microcomputed tomography, except for Zhai et al. [40], who reported only the histology for in vivo experiments. One study reported X-ray imaging [17], with descriptive results. Histology was reported in all studies. However, four studies presented a quantitative analysis of bone formation [18,20,39,41], while the other studies described histology findings [17,21,38,40]. Immunohistochemical analysis was reported by five studies, to evaluate the expression of several osteogenic markers, such as OCN [18,21,38,39]; BMP2 [21,38]; RUNX-2 [39]; BSP and DMP1 [21]; and angiogenic-related markers including VEGF, VE-cad [41], and CD31[18]. Immunofluorescence staining was also used to verify the expression of angiogenic-related markers such as CD31 [17,20,41] and endomucin [17]. Sequential fluorescence labeling was reported by two studies to evaluate the progression of bone regeneration by intraperitoneally injecting tetracycline, alizarin red, and calcitonin [18,20].

### 3.7. In Vitro and In Vivo Outcomes

The outcomes for osteogenic differentiation, angiogenesis, bone regeneration, and vascularization are summarized in Table 3 and Table 4.

#### 3.7.1. Osteogenic Differentiation

Osteogenic differentiation was performed in several studies by analyzing ALP activity as an early marker, ARS for mineral deposition, and the expression of osteogenesis-related genes. ALP activity [29,31,32,33,34,36,40] and mineral deposition [32,33,34,36,40] in cells treated with osteogenically induced exosomes were higher in most of the studies compared to non-osteogenic exosomes. The exception was reported by Wei et al. [35]; non-osteogenic exosomes were able to improve ALP activity and osteogenic marker expression (OPN, ALP, RUNX2, BMP2, and BMP7) in BMSCs cultured in OM compared to exosomes derived from 3, 7, and 14 days of osteogenic induction. Narayanan et al. [37] reported the expression of several osteogenesis-related genes and showed that 2- and 4-week osteogenically induced exosomes were able to upregulate when compared to untreated cells. However, when cultured in a 3D environment, cells treated with 4-week and non-osteogenic exosomes had similar results. Unfortunately, this study did not provide a comparison between exosome treatments.

The best day for collecting exosomes under osteogenic induction differed among studies. By analyzing the optimal exosome induction time, Li et al. [29] showed that exosomes derived from non-osteogenically induced ASCs could not enhance ALP activity either in OM or PM. However, exosomes derived from osteogenically induced ASCs significantly enhanced the ALP activity of BMSC culture in OM irrespective of the time span (2, 4, 7, and 14 days), but not in PM. Further analysis showed that ALP activity; mineral deposition; and RUNX2, ALP, and COL1A1 expression were higher in BMSCs with two-day induced exosomes cultured in OM compared to standard osteogenic medium alone. On the other hand, Wang et al. [36] showed that 3, 6, 9, and 12 days of osteogenic induction of hMSCs did not provide exosomes with the capacity to significantly induce the osteogenic differentiation of hMSCs when cultured in PM. However, 15-, 18-, and 21-day derived exosomes showed a higher mineral quantification of hMSCs. Yang et al. [34] only reported 14 days of the osteogenic induction of ASC-derived exosomes. The results showed that treatment improved ALP activity, gene and protein expression (RUNX and ALP), and mineral deposition of ASCs (cultured in OM) in comparison to non-osteogenic exosomes. Liu et al. [33] showed that exosomes from 3, and 14 days of osteogenic induction were able to enhance ALP activity in PM; when cultured in OM, 3-, 7-, and 14-day exosomes were able to enhance ALP activity. However, mineral deposition was significantly enhanced only by 14-day exosomes in PM, while in OM, 3-, 7-, and 14-day exosomes were capable of a significant improvement. Regarding gene expression, 14-day exosomes in PM and OM culture upregulated RUNX2, ALP, and osterix compared to non-osteogenic exosomes. Pishavar et al. [32] compared exosomes from osteogenically induced PSCs and BMSCs in the treatment of BMSCs cultured in OM. All EV-treated BMSCs showed higher levels of ALP activity at 14 days of culture in comparison to OM alone, except for non-osteogenic BMSC-derived exosomes. Calcium deposition was significantly higher after 21 day of induction by all EVs in comparison to OM alone, except for non-osteogenic PSC-derived exosomes. Xie et al. [31] reported only 7 days of osteogenically induced exosomes and showed qualitatively that they were able to enhance ALP activity and mineral deposition by DPSCs. The quantification of RUNX2, COL-1, and OCN showed that exosomes derived from 7-day induced DPSCs were able to upregulate their expression compared to non-osteogenic exosomes. Zhai et al. [40] evaluated exosomes from 4-, 10-, 15-, and 20-day osteogenically induced hMSCs in PM-cultured cells. Results showed that COL-1 expression was higher in 10-day-exosome-treated cells and OPN expression, ALP activity, and mineral deposition were higher in 10- and 15-day-exosome-treated cells—all compared to non-osteogenic exosomes. In summary, exosomes derived from late osteogenically induced MSCs can trigger osteogenic differentiation in cells treated in basal/growth media compared to early induced exosomes. However, when cells are cultured in osteogenic media treated with exosomes derived from osteogenically induced MSCs, a great improvement in ALP activity, osteogenic marker expression, and mineral deposition is seen.

Other conditioning methods of MSCs showed great results. Dexamethasone-activated BMSCs provided exosomes with the ability to improve the migration, proliferation, mineral deposition, and osteogenic marker expression (RUNX2, ALP, and OPN) of MC3T3-treated cells in comparison to non-conditioned exosomes [30]. Similarly, Ying et al. [18] reported that mutant BMSC-HIF1α-derived exosomes generated higher levels of BMSC proliferation, ALP activity, mineral deposition, and osteogenic marker expression (ALP, RUNX2, and COL1A1) compared to exosomes derived from non-mutant BMSCs. Li et al. [28] also used mutant BMSC-HIF1α-derived exosomes that resulted in the upregulation of OCN and ALP expression and enhanced mineral deposition by treated BMSCs. Exosomes derived from miR-375-overexpressing ASCs showed improvement in the ALP activity, mineral deposition, and osteogenic marker expression (RUNX2 and OCN) of BMSCS cultured in PM and OM compared to non-modified exosomes; ALP and COL1A1 were upregulated only when BMSCs were cultured in OM [38]. Huang et al. [21] performed genetic modification as well, by overexpressing BMP2 in BMSCs. The derived exosomes were able to upregulate the expression of BMP2, RUNX2, osterix, and BMP9 in BMSCs cultured in collagen sponge, but only when compared to untreated cells. A three-dimensional mechanical microenvironment was reported by Yu et al. [39]. The culture of PDLSCs in these conditions resulted in exosomes that were able to increase the mineral deposition and upregulate the expression of ALP, RUNX-2, OCN, and COL-1 in BMSCs when compared to exosomes derived from the non-mechanical 3D microenvironment.

#### 3.7.2. Angiogenesis

Four studies analyzed the effects of exosomes on HUVEC proliferation, migration, and tube formation [17,20,28,41]. DMOG-stimulated BMSC-derived exosomes were able to improve cell migration and tube formation but did not significantly improve cell proliferation compared to non-stimulated exosomes [20]. All exosome treatments evaluated by Liu et al. [17] and Liu et al. [41] were able to improve cell proliferation, migration, and tube formation; however, exosomes derived from hypoxic [17] and strontium-substituted-calcium-silicate-conditioned [41] cells showed significantly better results. In addition, the expression of VEGF was higher in HUVECs treated with hypoxic and strontium-substituted-calcium-silicate-conditioned exosomes. Exosomes from mutant-HIF-1α BMSCs were also able to promote angiogenesis when compared to exosomes derived from wild-type BMSCs [28]. In summary, all the above-cited conditioning methods were able to promote angiogenesis by increasing HUVEC proliferation, migration, and tube formation and were better than unconditioned exosomes.

#### 3.7.3. Bone Regeneration

Micro-CT and histology were the principal methods reported to describe bone regeneration in the studies. In all in vivo studies, treatment with exosomes derived from preconditioned MSCs led to a more advanced bone formation in comparison with unconditioned derived exosomes.

Among the in vivo studies, only one reported treatment with exosome derived from osteogenically induced MSCs [40], which contrasted with the in vitro studies, wherein standard osteogenic medium was the prevalent method of preconditioning. In this study, a more advanced bone formation was observed when the bone defects were treated with exosomes derived from 10- and 15-day osteogenically induced MSCs. The results described in these two groups included the presence of more Haversian canal-like structures, more bone cells, and more blood vessels. Additionally, more collagen formation and osteoblasts were observed, which were comparable to the MSC-seeded group. However, only descriptive analysis was presented in this study.

Other chemical additions to the MSC culture included DMOG [20] and strontium-substituted calcium silicate [41]. Both proved to be a good conditioning method for MSCs, generating exosomes with the ability to promote greater bone formation in a calvaria defect model [20] and a distal femur defect model [41] when compared to other exosome treatments.

In the category of genetic modifications, three studies reported exosome treatment in the rat calvaria defect model. Mesenchymal stem cells were modified to overexpress miR-375 [38] and BMP2 [21] and to express mutant HIF-1α [18]. Chen et al. [38] reported significantly greater bone formation in the defects treated with exosomes derived from miR-375-overexpressing MSCs compared to negative control exosomes. Compared to the blank group, both exosome types enhanced bone formation. The expression of OCN and BMP2 was analyzed by IHC and the range and intensity were higher in the miR-375-Exo group. Huang et al. [21] also observed a better performance of EVs derived from BMP2-overexpressing MSCs in bone formation compared to control EVs. The results of BMP2-EV-treated defects were comparable to recombinant human BMP2; however, the presence of a fatty marrow was observed in the latter but not the former. Additionally, the early expression of BMP2, BSP, DMP1, and OCN was more pronounced in BMP2 EVs, indicating a more rapid turn in the bone microenvironment. Similarly, Ying et al. [18] showed that mutant HIF-1α MSCs generate exosomes capable of effectively promoting bone regeneration. The association of exosomes with β-TCP enhanced bone formation compared to β-TCP alone, and exosomes carrying mutant HIF-1α were better than control exosomes. Furthermore, pronounced positive staining for OCN was observed in HIF-1α exosomes.

Hypoxic MSC-derived exosomes were reported by Liu et al. [17] and showed great improvement in bone formation. In a mice femur fracture model, a greater callus volume was observed in both exosome-treated groups; however, hypoxic exosomes showed a significant difference compared to exosomes derived from normoxia. A three-dimensional mechanical microenvironment was used to condition MSCs and was reported by Yu et al. [39] in an alveolar bone defect model. Exosomes derived from MSCs in these culture conditions were able to enhance the new bone area and volume as well as the expression of RUNX-2 and OCN.

Collectively, the studies’ results show that exosomes from both conditioned and unconditioned MSCs can enhance bone formation compared to untreated defects. However, the conditioning methods of the MSCs used in all the reported studies generated exosomes with superior potential to promote bone regeneration when compared to exosomes derived from unconditioned MSCs.

#### 3.7.4. Vascularization

Four studies reported a vascularization assessment in vivo, mostly with microfil perfusion by micro-CT analysis and immunohistochemistry. Ying et al. [18] showed that exosomes derived from mutant HIF-1α cells promoted a greater vessel area, vessel number, and CD31 expression in a rat calvaria defect model. Similarly, Liu et al. [17] showed that hypoxic MSC-derived exosomes could enhance vessel volume, vessel number, and the expression of CD31 and endomucin in a mice femur fracture model. In a calvaria defect model, DMOG-stimulated MSC-derived exosomes promoted a significantly greater vessel area and expression of CD31 [20]. The expression of CD31, VEGF, and VE-cad was significantly enhanced in a distal femur defect model treated with exosomes derived from strontium-substituted-calcium-silicate-stimulated MSCs.

### 3.8. Secondary Outcomes

Twelve studies reported secondary outcomes, related mostly to signaling pathways and miRNA profiles (Table 5).

The upregulation of several miRNAs was observed in exosomes from different stages of osteogenic induction compared to exosomes from undifferentiated MSCs. Namely, miR-31-3p/5p, miR-10b-5p [36], miR-122-5p, miR-142-5p, miR-25-3p, miR-192-5p [33] miR-130a-3p, miR-30b-5p, miR-34a-5p, miR-324-5p, miR-378f [34], miR-186, miR-210, miR-181c-5p [32], Hsa-miR-146a-5p, Hsa-miR-503-5p, Hsa-miR-483-3p, and Hsa-miR-129-5p [40] were upregulated in exosomes derived from 10- [40], 14- [33,34], 15- [40], and 21-day osteogenically induced MSCs [32,36]. These miRNAs were predicted to be involved in bone formation signaling pathways, such as Wnt [32,34,36], MAPK [32,33,34,36,40], AMPK, insulin [33,36], Hippo [32,36], TGF-β [32,34,36], and PI3K/Akt [36,40].

One study showed that the pro-osteogenic effects of exosomes derived from 0-day osteogenically induced MSCs (undifferentiated cells) were mediated by protein phosphorylation [35]. An increase in the phosphorylation of proteins implicated in bone metabolism (STAT6, GSK-3α/β, STAT5b, and STAT5a/b) was observed in a BMSC culture. Furthermore, SMAD-related genes (SMAD4 and BMPR2), related to the TGF-β signaling pathway, were upregulated.

Three studies provided secondary outcomes related to angiogenesis [17,20,41]. Exosomes from DMOG-stimulated MSCs were able to downregulate the expression of PTEN, and its deficiency is related to the increased migration and invasion of HUVECs [20]. Liu et al. [17] reported the upregulation of miR-126 in exosomes derived from hypoxic MSCs, and they showed that the knockdown of miR-126 inhibited the ability of these exosomes to mediate proliferation, migration, and angiogenesis in vitro and in vivo. Similarly, the stimulation of MSCs with strontium-substituted calcium silicate generated exosomes with upregulated miR-146a, and its inhibition led to diminished migration, tube formation, and VEGF and ANG1 expression in HUVECs [41].

The three-dimensional mechanical microenvironment upregulated mir-10a-5p and mir-10b-5p and downregulated mir-212-3p in exosomes; however, signaling pathway prediction was not reported [39]. The overexpression of BMP2 in BMSCs generated exosomes that were able to trigger SMAD 1/5/8 phosphorylation [21].

A different analysis was reported by Xie et al. [31], whereby they searched for changes in circRNA expression during the osteogenic differentiation of DPSCs. The levels of circLPAR1 (hsa_circ_0003611) increased in exosomes derived from 7-day induced MSCs, and it was predicted to bind to hsa-miR-31, a miRNA that showed a significant inhibitory effect against osteogenic differentiation. Both the downregulation of hsa-miR-31 and the upregulation of circLPAR1 improved osteogenic differentiation.

### 3.9. Risk of Bias Assessment

**Figure 2:**
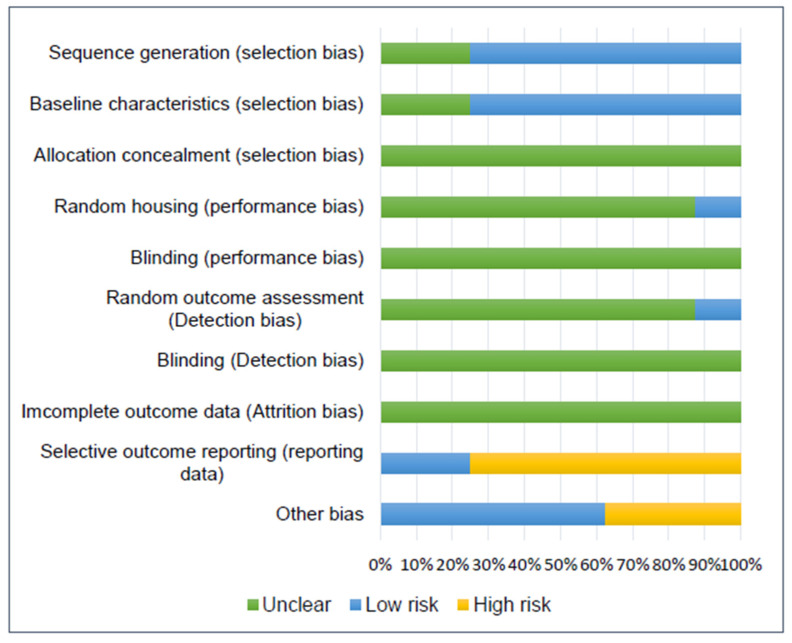
Risk of bias chart for assessing the methodological quality of the 8 papers reporting in vivo studies included in this systematic review.

The studies included for in vivo experiments were assessed for their risk of bias by the SYRCLE tool, and the results are illustrated in Figure 2.

Regarding selection bias, six studies reported the randomization of group allocation and were assigned a low risk, but the randomization process was not specified [17,18,20,38,40,41]. Two studies did not describe the baseline characteristics of the animals; therefore, they were assigned an unclear risk [17,21]. Allocation concealment was not mentioned in any study included; thus, they were assigned an unclear risk.

Poor information was reported by the studies regarding performance, detection, and attrition bias (random housing, research blinding, random outcome assessment, outcome blinding, and incomplete outcome data); therefore, an unclear risk was assigned in most cases. Only one study described the housing conditions and was classified as low risk for random housing [38]. Furthermore, only one study reported the random outcome assessment and was classified as low risk, although it did not mention the randomization process [18].

Six studies were classified as having a high risk of bias for selective outcome reporting [20,21,38,39,40,41]. They all failed to clearly mention the methodology for histology, histomorphometry, or IHC results, e.g., not mentioning the software used for quantification or the methodology for qualitative or semi-quantitative results. Furthermore, Zhai et al. [40] described in the Section 2 seven groups for in vivo experiments; however, in the Section 3 only a few of them were reported.

Other risks of bias included a lack of clear information regarding treatment distribution along with the defects and animals [40,41], e.g., the existence of a blank group without indication as to whether it was a separate group or the contralateral limb [40] or if one animal received more than one treatment in different limbs [41]. Furthermore, one study reported a very low number of animals included in the experiment (*n* = 8), resulting in only one animal for time point/treatment analysis [39].

## 4. Discussion

This review showed that all approaches used to conditioned MSCs enhanced the therapeutic ability of exosomes for bone regeneration. However, what do these approaches have in common?

In general, the conditioning or priming of MSCs are performed to mimic or magnify a response that they would have in the natural microenvironment and produce a targeted message. However, for MSCs to promote osteogenesis, angiogenesis, immunomodulation, and several other effects in the natural in vivo microenvironment, the niche is involved. The three-dimensional space that MSCs are inserted into provides cell-to-cell contact and biochemical and biomechanical signals which will determine the function and actions of these cells [42]. Therefore, the functions of exosomes will be affected by the niche and the microenvironment into which the exosome-secreting cells are inserted.

In this sense, the osteogenic induction method is the more obvious approach. The addition of several chemicals in the culture medium leads to a commitment of the cells towards the osteogenic lineage. Once the cells are committed to that lineage, they start producing targeted information, which is partially packed within their respective EVs [32,33,34,36,40]. When MSCs in a mimetic osteogenic microenvironment (cultured in OM) were submitted to exosomes derived from osteogenically induced MSCs, an enhancement was observed in the osteogenic differentiation, showing that these cells were able to assimilate the information carried by the exosomes and generate a response. When the cells were not cultured in a mimetic osteogenic microenvironment (PM), only the exosomes from highly differentiated cells were able to barely generate a response towards osteogenic commitment [29,33,36,40]. This shows that the effects of exosomes will depend on the microenvironment they are applied to. However, in the in vivo scenario, an interplay of events known as the inflammatory phase occurs at the moment of injury, wherein the cascade of events will begin [1], which strongly differs from the in vitro environment. Only one study included in this review applied exosomes from osteogenically induced MSCs in a bone defect model [40], and although the miRNA profile indicated differently expressed miRNAs that could be connected to the improvement observed in the in vivo results, only a descriptive analysis was performed of the histology findings. Therefore, there is still a lack of evidence regarding the benefits of exosomes from osteogenically induced MSCs in bone regeneration and how they are involved in the extensive events occurring at the beginning of the healing phase.

Dexamethasone is routinely used as part of the osteogenic medium components to differentiate MSCs. Zhao et al. [30], by only using a low concentration of dexamethasone to enhance the commitment of MSCs, showed that the derived microvesicles could promote the osteogenic differentiation of preosteoblasts even more than the standard osteogenic medium. However, there is still a lack of evidence for the effects of EVs derived from this type of conditioned method in vivo, since the reported in vivo experiments did not include a control group of unconditioned exosomes. The content of exosomes derived from this type of conditioning should be further analyzed, along with the effects of the treatment in bone defect models. Furthermore, dexamethasone in vivo is related to pathological conditions such as osteoporosis [43], therefore reinforcing the need for a more detailed analysis.

Two studies focused on the genetic modification of MSCs by overexpressing two components that play a role in bone regeneration: BMP2 [21] and miR-375 [38]. Bone morphogenetic proteins belong to the TGF-β superfamily and have an important osteoinductive role in the bone microenvironment [44]. Specifically, BMP2 is produced by osteoblasts and osteoprogenitors [1] and initiates the repair cascade, promoting the differentiation of MSCs in osteoblasts or chondroblasts [44,45]. The overexpression of BMP2 in BMSCs generated EVs that stimulated bone formation significantly more than control EVs and were comparable to rhBMP2 treatment. However, instead of forming bone with a fatty marrow, as in the rhBMP2 group, EVs derived from BMP2-overexpressing BMSCs promoted a dedicated intramembranous bone regeneration process [21], a well-determined part of bone regeneration. This strategy for MSC conditioning is promising for bone regeneration, due to the well-established role of BMP2.

MicroRNAs are a class of small, non-coding RNAs that negatively regulate gene expression at the mRNA level [46]. Their biological functions have been discovered throughout miRNA-knockout models and overexpression experiments [47] and include differentiation and development, metabolism, proliferation, apoptotic cell death, viral infection, and tumorigenesis [48]. The role of miRNA-375 in the osteogenic differentiation of ASCs was confirmed by Chen and coworkers [19], and the exosomes derived from miRNA-375-overexpressing ASCs enhanced bone formation in vivo [38]. Thus, miRNA in osteogenesis is a field that could unravel potential targets for bone regeneration.

Lineage commitment is not the only thing needed for MSCs to exert their positive effects. For example, to activate MSCs in a fracture microenvironment, cells present in the early inflammatory phases will send signals, such as growth factors and pro-inflammatory cytokines [49]. Then, upon activation, MSCs will also function as secretory cells, including angiogenic factors [5,50,51]. Angiogenesis and osteogenesis are tightly related, and bone health is entirely dependent on vascular supply [52]. Five articles included in this review analyzed the role of MSC-conditioning methods in angiogenesis and bone healing [17,18,20,28,41].

Mesenchymal stem cells in natural physiological conditions are exposed to a low concentration of oxygen, ranging from 2 to 9% (or lower), while for in vitro cultures of MSCs, the oxygen concentration is around 21% [53]. Studies have demonstrated that culturing MSCs in hypoxic conditions improves their therapeutic effects upon direct and indirect application in several scenarios, such as myocardial repair [54,55], cerebral ischemia [56,57], skin wound healing [58,59], and bone healing [17,60,61]. Hypoxically conditioned MSCs produced exosomes that enhanced proliferation, migration, and tube formation by HUVECs, and the bone formation and vascularization of a mice fracture model; additionally, the protein concentration of these exosomes was higher than unconditioned ones, indicating that hypoxia can improve MSC-derived exosome yield. Interestingly, the mechanisms behind these results are related to the enriched levels of miR-126, which was possibly mediated by hypoxia-inducible factor-1α (HIF-1α) [17].

In hypoxic conditions, the HIF-1α is upregulated. Genes related to angiogenesis and osteogenesis, such as VEGF and RUNX2, are regulated by this transcription factor [62,63,64]. Therefore, genetic modification was performed to generate mutant-HIF-1α-modified BMSCs, and in vitro results showed that exosomes derived from these cells greatly improved HUVEC proliferation, migration, and tube formation [28]. In a calvarial defect model, significantly greater bone and neovascular formation was observed when treated with exosomes derived from mutant-HIF-1α BMSCs. It was also shown that these exosomes could promote proliferation, osteogenic differentiation, and RUNX expression by BMSCs [18]. When taking into consideration the microenvironment of a fracture site, HIF-1α kick-starts several other mechanisms, and it is an ideal target and good approach for bone regeneration.

Also aiming at the angiogenic properties of MSCs, Liang and coworkers [20] conditioned these cells using DMOG, instead of genetically modifying them or culturing them in hypoxia. The HIF-1α transcription factor remains stable only under hypoxic conditions [18], and DMOG is an angiogenic molecule that inhibits the degradation of HIF-1α, stabilizing its expression by cells under normal oxygen conditions [65,66]. This approach has shown improvements in neurodegenerative diseases [67], cardiac ischemia [68], and bone regeneration [69,70]. The exosomes derived from DMOG-stimulated MSCs significantly promoted bone and vessel formation in a calvarial defect model and HUVEC proliferation, migration, and tube formation. Interestingly, the in vitro results were related to the downregulation of PTEN in HUVECs treated with DMOG exosomes, which lead to the activation of the AKT/mTOR pathway [20].

The capacity to both promote osteoblast and inhibit osteoclast functions led to the extensive use of strontium in bone research [71]. Strontium (Sr) is a bone-seeking element, and the introduction of strontium ranelate in clinical trials showed its role in preventing fractures in osteoporotic patients [72]. The mechanism by which Sr acts in an organism relies on its actions towards cellular targets similar to those of calcium (Ca), and so it interacts with signaling pathways related to calcium-sensing receptors [73]. Studies have shown that the partial substitution of Ca by Sr in ceramic- and cement-based biomaterials leads to an upregulation in the expression of osteogenesis- and angiogenesis-related genes [74] and an improvement in osteointegration [75]. The in vitro stimulation of BMSCs by strontium-substituted calcium silicate (Sr-CS) upregulated the expression of RUNX2, BMP-2, VEGF, and ANG1—all genes that are strongly related to bone regeneration. The derived exosomes of Sr-CS-stimulated BMSCs were able to promote the in vitro angiogenesis of HUVECs and angiogenesis and bone formation in vivo by differentially expressed miR-146a [41]. Thus, beyond the application of Sr in vivo in association with biomaterials, its use to condition MSCs and their derived exosomes is a viable and promising approach for bone regeneration. Further studies in other bone defect models should elucidate even more the therapeutic effects of Sr-CS.

The bone fracture site is filled with cells and biological factors that are organized in a timed and spatially coordinated performance, and their response to the mechanical stimuli will guide the migration, proliferation, and differentiation of progenitor cells [76]. The mechanical stimuli can act by external or intracellular forces that generate changes in the expression of several biological factors by a mechanism called mechanical transduction [77]. Using collagen as a three-dimensional environment with applied mechanical strains, Yu and coworkers [39] cultured PDLSCs and extracted their exosomes. The derived exosomes enhanced mineralization and upregulated the expression of ALP, RUNX2, OCN, and COL-1 by BMSCs in vitro. Furthermore, the exosomes were able to improve bone formation in a rat alveolar bone defect, although the number of animals was very small. A 3D mechanical environment is an option for the enhancement of the therapeutic effects of MSC-derived exosomes; however, more in vivo studies should further elucidate its mechanism.

**Figure 3:**
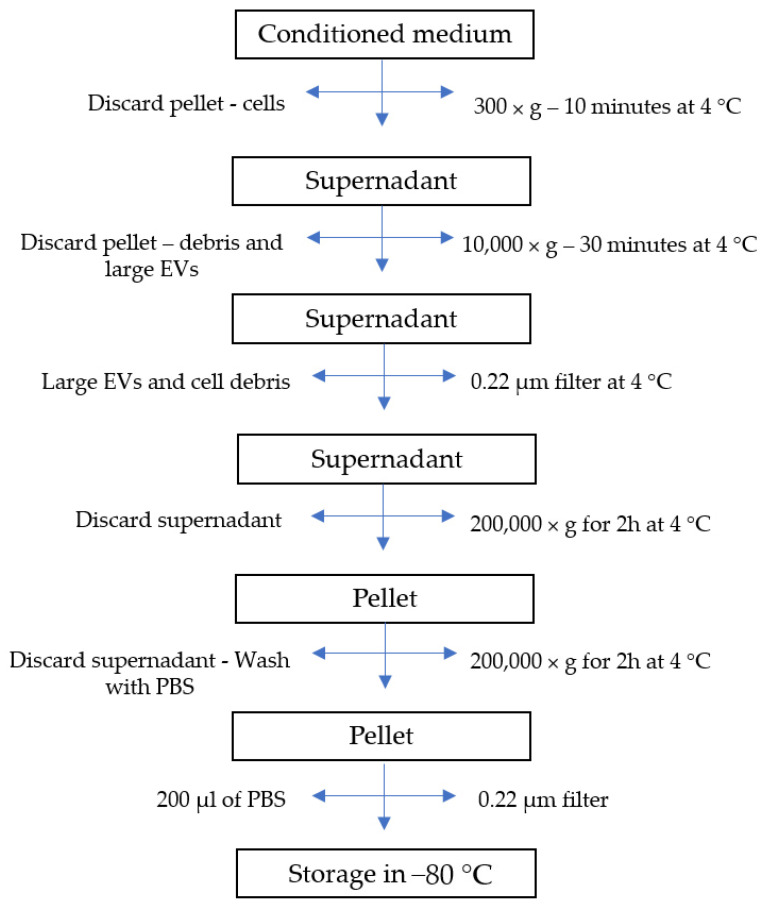
Flow diagram of extracellular vesicle isolation process.

**Figure 4:**
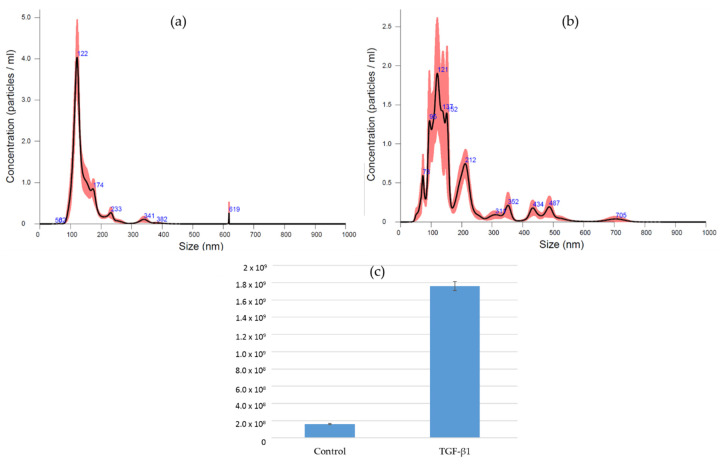
Purified EVs suspended in PBS were subjected to nanoparticle tracking analysis. (**a**) EVs purified from CM of control cells. (**b**) EVs purified from CM of TGF-β1-treated cells. (**c**) Particle concentration of purified EVs from control and TGF-β1-treated cells (dilution factor 1:25).

The several conditioning methods reported in this review provided results that place EVs in the race for cell-free bone regeneration therapies. The authors’ current research involves the preconditioning of canine umbilical cord perivascular cells with transforming growth factor (TGF)-β1 and the characterization of their derived EVs. This growth factor is part of the TGF-β superfamily, which is known to play an important role during bone repair [78]. Furthermore, it has been documented that this growth factor improves the migration of bone-marrow-derived MSCs [79] and optimizes the differentiation of these cells toward the osteogenic lineage [80]. Preliminary results showed that EVs were successfully isolated from the conditioned medium (CM) by differential centrifugation and ultracentrifugation (Figure 3). Nanoparticle tracking analysis (Nanosight) demonstrated the isolation of EVs with a mode size of 121.6 and 120.2 nm for control and TGF-β1 EVs, respectively. Additionally, the particle distribution showed the superior production of vesicles by the TGF-β1 group (1.76 × 109 +/− 5.13 × 107 particles/mL) in comparison to the control group (1.61 × 108 +/− 7.58 × 106) (Figure 4). The low yield of EVs isolated from the conditioned medium is a limiting aspect for their clinical application; therefore, it is an important factor to consider when choosing a preconditioning method.

Strong findings were reported by the majority of studies, strengthening the urge for new and complementary research, such as: (i) the well-described conditioning and culturing methods; (ii) the well-characterized EVs; (iii) in vitro assays well-described with concise and complete results; (iv) in vivo bone regeneration well-reported by microcomputed tomography; and (v) the complementary analysis of miRNA profiles, signaling pathways, and gene expression. However, the in vivo studies were conducted only in small animals with varied bone defect models, consequently leaving a lack of clear evidence. Larger animals with critical bone defects or non-union models should be the next step to evaluate the conditioning methods of MSCs and their derived exosomes.

Despite the evidence of the therapeutic potential of EVs derived from conditioned MSCs, the heterogeneity of the cell sources, EV concentrations, and the scaffolds used limits the evidence supporting a particular conditioning method as the best option for bone regeneration so far. Additionally, the reporting of outcomes could be better addressed in further studies, advocating for the quantitative analysis of standardized methods for in vivo bone regeneration assessment.

Limitations were found mostly regarding the unclear risk of bias in most of the SYRCLE tool’s domains. The lack of reporting information pertaining to allocation concealment, housing randomization/conditions, blinding of performance, and blinding outcome assessment meant that the studies were at risk of bias. Furthermore, the lack of reports of the methodology, i.e., the specific method of assessment and how it was performed, diminished the strength of the studies’ evidence. For further studies focusing on the therapeutic potential of EVs derived from conditioned MSCs, this review suggests critically analyzing the best conditioning method according to the specific goal of the study, including the most suitable cell source, an approach that increases the yield of EVs, diminishes the concentration for clinical application, and is financially viable. Additionally, following the guidelines for studies of extracellular vesicles [81] and the ARRIVE guidelines for reporting in vivo studies [82] can improve the reproducibility of a study and translate the science from bench to bedside.

## 5. Conclusions

This review systematically assessed the application of EVs derived from conditioned MSCs in bone regeneration in vitro and in vivo, evidencing that different conditioning methods do improve the therapeutic effects of MSC-derived EVs for bone regeneration. These findings, despite being promising, still rely on a few heterogeneous studies (in terms of the conditioning approaches, animal and defect models, and therapeutic dosages) and still need to be addressed in larger animal models for further clinical application.

## Figures and Tables

**Table 1:** Conditioning methods.

Type of Conditioning	Conditioning Method	Reference
Chemical induction	Standard osteogenic medium	[29,31,33,36,40]
	Dimethyloxalylglycine	[20]
	Dexamethasone	[30]
	Strontium-substituted calcium silicate ceramics	[41]
Chemical induction + culture conditions	Standart osteogenic medium + serum deprivation	[32,34,35,37]
Genetic modification	miR-375-overexpressing ASCs	[38]
Genetic modification + culture conditions	BMP2-overexpressing BMSCs + serum deprivation	[21]
	Mutant HIF-1α-modified BMSCs + serum deprivation	[18,28]
Culture conditions	Hypoxia	[17]
	Three-dimensional mechanical microenvironment	[39]

ASCs, adipose-tissue-derived stem cells; BMP2, bone morphogenetic protein 2; BMSCs, bone marrow mesenchymal stem cells; HIF, hypoxia-inducible factor.

**Table 2:** Methodology information for EV isolation.

EV Source/Origin	Type of EV	Isolation Method	Characterization Method	EV Markers	Size Distribution	Reference
Human ASCs	Exosomes	Ultracentrifugation	TEM, NTA, WB	CD63, CD9	33–177 nm	[29]
Human ASCs	Exosomes	Ultracentrifugation	TEM, NTA, WB	CD9, CD63	~75 nm	[38]
Human ASCs	Exosomes	Ultracentrifugation	TEM, WB	TSG101, CD9	30–150 nm	[34]
Human BMSCs	Exosomes	ExoQuick TC isolation kit, System Biosciences	TEM	NR	NR	[37]
Human BMSCs	Exosomes	Ultracentrifugation	TEM, WB, qNano platform	CD9, CD63, GM130, TSG101	30–100 nm	[20]
Human BMSCs	Exosomes	Ultracentrifugation	TEM, NTA	NR	~100 nm	[35]
Human BMSCs	Extracellular vesicles	ExoQuick TC isolation kit, System Biosciences	TEM, NTA, immunoblotting	CD63, CD9	100–150 nm	[21]
Human BMSCs and Human PSCs	Extracellular vesicles	Ultrafiltration + ultracentrifugation	TEM, DLS	NR	BMSC: ~137–262 nm PSC: ~130–202 nm	[32]
Human DPSCs	Exosomes	Ultracentrifugation	TEM, NTA, flow cytometry	CD63, CD81	20–120 nm	[31]
Human MSCs *	Exosomes	Ultracentrifugation	TEM, NTA	NR	30–150 nm	[36]
Human MSCs *	Exosomes	ExoQuick TC isolation kit, System Biosciences	AFM, TEM, NTA, WB	CD63	~143 nm	[40]
Human PDLSCs	Exosomes	ExoQuick TC isolation kit, System Biosciences	TEM, NTA, WB	CD63, CD81	30–200 nm	[39]
Human PDLSCs	Exosomes	Ultracentrifugation	TEM, NTA, WB	CD63, TSG101	~120 nm	[33]
Human UCMSCs	Exosomes	Ultrafiltration + sucrose + ultracentrifugation	TEM, NTA, WB	TSG101, CD9, CD63, CD81	50–150 nm	[17]
Rabbit BMSCs	Exosomes	Exosome isolation kit, Invitrogen	TEM, WB, qNano Platform	CD9, CD63, CD81	75–150 nm	[28]
Rat BMSCs	Microvesicles	Differential centrifugation **	TEM, NTA, flow cytometry	CD90	100–400 nm	[30]
Rat BMSCs	Exosomes	Ultrafiltration + sucrose + ultracentrifugation	TRPS, WB	CD9, CD63, CD81	50–150 nm	[18]
Rat BMSCs	Exosomes	Ultracentrifugation and ultrafiltration	TEM, NTA, WB	CD9, CD63, CD81	~130 nm	[41]

AFM, atomic force microscopy; ASCs, adipose-derived mesenchymal stem cells; BMSCs, bone marrow mesenchymal stem cells; DLS, dynamic light scattering; DPSCs, dental pulp stem cells; EV, extracellular vesicle; MSCs, mesenchymal stem cells; NTA, nanoparticle tracking analysis; NR, not reported; PDLSCs, periodontal ligament stem cells; PSCs, placental stem cells; TEM, transmission electron microscopy; TRPS, tunable resistive pulse sensing; UCMSCs, umbilical cord mesenchymal stem cells; WB, Western blotting. * Cell source not specified; ** fewer steps and lower g.

**Table 3:** Summary of in vitro methods and outcomes.

MSC Conditioning Method	Culture Method for Control EVs	Exposure to EVs	Treatment/Groups	Analysis	EV Concentration	Outcome	Ref.
Human ACSs conditioned with osteogenic medium for 2, 4, 7, and 14 days (xd-Exo)	Human ASCs cultured with normal medium without stimuli (0d-Exo)	Human BMSCs	Cells cultured in OM or PM with 0d-Exo, 2d-Exo, 4d-Exo, 7d-Exo, and 14d-Exo	ALP activity	10, 25, or 50 μg/mL	↑ in cell differentiation in OM by osteogenically induced exosomes at 25 μg/mL exosomes.	[29]
Human ASCs conditioned with osteogenic medium for 14 days + serum-free medium for 24 h (Exo-D14)	Human ASCs cultured with serum-free medium for 24 h (Exo-D0)	Human ASCs	Exo-D0 and Exo-D14 (both cultured in OM); negative control (PM); positive control (OM)	ALP activity, ARS, osteogenic markers	20 µg/mL	↑ in cell differentiation in Exo-D14-treated ASCs compared to Exo-D0.	[34]
Human BMSCs conditioned with osteogenic medium for 2 and 4 weeks + serum-free medium for 24 h (two- or four-week exosomes)	Human BMSCs cultured in serum-free medium for 24 h (regular exosomes)	Human BMSCs	2D culture: two-week exosomes; four-week exosomes; regular exosomes; control. 3D type I collagen: four-week exosomes; regular exosomes; control	Pro-osteogenic gene analysis	NR (exosomes isolated from 5 × 10^5^ cells)	2D culture: upregulation of pro-osteogenic genes by two- and four-week exosomes. 3D culture: both four-week exosomes and regular exosomes upregulate pro-osteogenic genes. This article did not compare the results between the three types of exosomes.	[37]
Human BMSCs conditioned with osteogenic medium for 3, 7, or 14 days + serum-free medium for 12 h (xd-Exo)	Human BMSCs cultured with serum-free medium for 12 h (0d-Exo)	hBMSCs RAW 264.7	Human BMSCs cultured in OM treated with 0d-Exo, 3d-Exo, 7d-Exo, and 14d-Exo	ALP activity, osteogenic markers	10 µg exosome protein	↑ in cell migration by 3d-Exo, 7d-Exo, and 14d-Exo. ↑ in ALP activity, OPN, ALP, RUNX2, BMP2, and BMP7 when treated with 0d-Exo. ↓ in inflammation by 0d-Exo, 3d-Exo, and 7d-Exo.	[35]
			Human BMSCs treated with 0d-Exo, 3d-Exo, 7d-Exo, and 14d-Exo	Cell viability, cell migration	Cell viability: NR cell migration:10 µg exosome protein		
			RAW 264.7 treated with 0d-Exo, 3d-Exo, 7d-Exo, and 14d-Exo	Cytokine expression	10 µg exosome protein		
Human BMSCs and PSCs conditioned with osteogenic medium for 7 and 21 days + serum-free medium for 72 h (Dx)	Human PSCs and BMSCs cultured in serum-free medium for 72 h (D0)	Human BMSCs	Cells cultured in OM treated with D0, D7, and D21 derived from PSCs and BMSCs	ALP activity, ARS	10 μg/mL	↑ in cell differentiation by both BMSC- and PSC-derived exosomes, mainly by 21-day osteogenically induced exosomes.	[32]
Human DPSCs conditioned with osteogenic medium for 7 days (Exo7)	Human DPSCs cultured in serum-free medium for 48 h (Exo0)	Human DPSCs	Cells cultured in PM treated with Exo7 and Exo0 positive control (PC; OM), negative control (NC; PM)	ALP activity, ARS, osteogenic markers	NR	↑ in cell differentiation and expression of RUNX2, COL-1, and OCN by Exo7.	[31]
Human MSCs conditioned with osteogenic medium for 3, 6, 9, 12, 15, 18, and 21 days (Exo-Dx)	Human MSCs in passage 6, cultured in normal medium without stimuli (Exo-P6)	HumanMSCs	Cells culture in PM with human fibronectin-coated plates treated with exo-P6, D3, D6, D9, D12, D15, D18, and D21; negative control at day 0 (NCtrl-D0), NCtrl-D14, and NCtrl-D21	ALP activity, ARS	NR	↑ in cell differentiation by mid-to-late osteogenically induced exosomes (Exo-D15, Exo-D18, and Exo-21).	[36]
Human MSCs conditioned with osteogenic medium for 4, 10, 15, and 20 days (Exo-Dx)	Human MSCs cultured in normal medium without stimuli (Exo-D0)	Human MSCs	Cells cultured in PM treated with Exo-D0, Exo-D4, Exo-D10, Exo-D15, and Exo-D20	ALP activity, ARS, osteogenic markers (immunofluorescence staining)	100 μL of 1.0 × 10^13^ particles per mL	↑ in cell differentiation by Exo-D10 and Exo-D15 compared to Exo-D0.	[40]
Human PDLSCs conditioned with osteogenic medium for 3, 7, and 14 days (Exo-Dx)	Undifferentiated human PDLSCs (Exo-NC)	Rat BMSCs	Cells cultured in OM or PM treated with Exo-D3, Exos_D7, Exos-D14, Exos-NC, and PBS	ALP activity, ARS, osteogenic markers	50 μg/mL	↑ in cell differentiation by Exo-D3 and Exo-D14 in PM. ↑ in cell differentiation by Exo-D3, Exo-D7, and Exo-D14 in OM.	[33]
Human BMSCs conditioned with dimethyloxaloylglycin (DMOG, 1000 µM) for 48 h (DMOG-MSC-Exos)	Human BMSCs cultured in normal medium without stimuli (MSC-Exos)	HUVECs	MSC-Exos, DMOG-MSC-Exos, or an equivalent volume of PBS	Cell proliferation, migration, and tube formation	50 µg/mL	↑ in cell migration and tube formation by DMOG-MSC-Exos with no difference in cell proliferation.	[20]
Rat BMSCs conditioned with Dexamethasone (10^−8^, 10^−7^, 10^−6^ M) for 48 h (DXM-MV)	Rat BMSCs cultured in normal medium without stimuli (n-MV)	MC3T3	Cells cultured in PM with n-MV or DXM-MV (10^−8^, 10^−7^, 10^−6^ M)	ARS, osteogenic markers	NR	↑ in cell differentiation, migration, and proliferation by DXM-MVs.	[30]
Rat BMSCs stimulated by strontium-substituted calcium silicate ceramics for 48 h (Sr-CS-Exo)	Rat BMSCs cultured in normal medium without stimuli (Exo)	HUVECs	Cells treated with PBS, Exo (without stimuli), β-TCP-Exo, CS-Exo, and Sr-CS-Exo	Cell proliferation, cell migration, tube formation, and angiogenesis-related gene expression	Cell proliferation: 50 or 100 μg/mL; cell migration, tube formation, and angiogenesis-related gene expression: NR	↑ in cell proliferation, migration, tube formation, and gene and protein expression for VEGF and ANG1 by the Sr-CS-Exo.	[41]
miR-375-overexpressing human ASCs (Exo (miR-375))	Human ASC-overexpressing control vector (Exo (NC))	Human BMSCs	Cells culture in OM or PM treated with Exo (miR-375) and Exo (NC)	ALP activity, ARS, osteogenic markers, cell proliferation	Exo (miR-375): 50 μg/mL; Exo (NC): NR	↑ in cell differentiation and RUNX2 and OCN expression by Exo(miR-375) in PM and OM. ALP and COL1A1 were upregulated by Exo(miR-375) only in OM.	[38]
BMP2-overexpressing human BMSCs + serum-free medium for 24 h (BMP2 FEEs)	Human BMSCs cultured with serum-free medium for 24 h (control EV)	Human BMSCs	BMSCs cultured in collagen sponge with PM treated with BMP2 FEEs or untreated group (PBS)	Osteogenic markers *	1 × 10^8^ EV particles	BMP2 FEEs increased the expression of BMP2, RUNX2, osterix, and BMP9 in 3D BMSC culture compared to untreated cells. There was no control EV treatment in this analysis.	[21]
			Cells treated with control EVs and BMP2 FEEs; rhBMP2 (positive control)	SMAD1/5/8 phosphorylation	6 × 10^6^ EVs for every 30,000 HMSCs.		
Mutant HIF-1α-modified rabbit BMSCs (BMSC-Exos^MU^) + serum-free medium for 24 h	Rabbit BMSCs modified by wild-type HIF α (BMSC-Exos^WT^) + serum-free medium for 24 h	Rabbit BMSCs	BMSC-Exos^MU^ BMSC-Exos^WT^	ARS, ALP activity, osteogenic markers	80 μg/mL	↑ in cell differentiation and expression of OCN and ALP by BMSC-Exos^MU^. ↑ in HUVEC proliferation, migration, and tube formation by BMSC-Exos^MU^.	[28]
		HUVECs	BMSC-Exos^MU^ BMSC-Exos^WT^	Cell proliferation, cell migration, and tube formation	80 μg/mL, 40 μg/mL, and 20 μg/mL per group		
Mutant HIF-1α-modified rat BMSCs + serum-free medium for 48 h (BMSC-Exos-HIF1 α)	Culture method not reported (BMSC-Exos)	Rat BMSCs	Cells cultured in PM treated with: BMSC-Exos-HIF1α, BMSC-Exos, and control	Cell proliferation	200 μg/mL	↑ in cell differentiation; proliferation; and expression of ALP, RUNX2, and COL1-a1 by BMSC-Exos-HIF1α.	[18]
			Cells cultured in OM and treated with BMSC-Exos-HIF1α, BMSC-Exos, and control	ALP activity, ARS, osteogenic markers	200 μg/mL		
Human PDLSCs cultured in three-dimensional microscale magnetically stretched collagen hydrogels (SM-Exo)	Human PDLSCs cultured in 3D culture (Exo)	Human BMSCs	Cells cultured with PBS, SM-Exo, and Exo	Cell proliferation, cell migration	100 μg/mL	↑ in cell differentiation; proliferation; migration; and ALP, RUNX-2, OCN, and COL-1 expression by SM-Exo.	[39]
			Cells cultured in OM treated with PBS, SM-Exo, and Exo	Osteogenic markers, ARS	100 μg/mL		
Human UCMSCs conditioned with hypoxia (Hypo-Exos)	Human UCMSCs cultured in normoxia (Exos)	HUVECs hFOB 1.19	Cells treated with PBS, Exos, or Hypo-Exos	Cell proliferation, migration, tube formation, and angiogenesis-related genes	Cell proliferation, migration, and tube formation: 100 μg/mL; angiogenesis-related genes: NR	↑ in HUVEC proliferation, migration, tube formation, and VEGF expression by Hypo-Exos. ALP, COLA1, and OCN expression in hFOB 1.19 did not differ among groups.	[17]
				Osteogenic markers	NR		

ALP, alkaline phosphatase; ANG1, angiopoietin 1; ARS, alizarin red staining; ASCs, adipose-derived mesenchymal stem cells; BMP, bone morphogenetic protein; BMSCs, bone marrow mesenchymal stem cells; COL-1/COL1-A1, collagen type 1; DPSCs, dental pulp stem cells; DXM, dexamethasone; EVs, extracellular vesicles; Exo, exosome; FEEs, functionally engineered EVs; hFOB, human fetal osteoblastic cell line; HUVECs, human umbilical vein endothelial cells; IL, interleukin; MC3T3, osteoblast precursor cell line; MSCs, mesenchymal stem cells; MU, mutant; MV, microvesicles; NC/NCtrl, negative control; NR, not reported; OM, osteogenic medium; OPN, osteopontin; PBS, phosphate-buffered saline; PDGFA, platelet-derived growth factor A; PDLSCs, periodontal ligament stem cells; PM, normal growth medium; PSCs, placental stem cells; RUNX-2, Runt-related transcription factor 2; Sr-CS, strontium-substituted calcium silicate; UCMSCs, umbilical cord mesenchymal stem cells; WT, wild type; ↑, upregulated; ↓, downregulated). * Article did not report a control group of EVs in this analysis.

**Table 4:** Summary of in vivo methods and outcomes.

Bone Defect Model	Animal Model	Treatment Groups	Concentration of EVs	Scaffold/Vehicle	Time Point Analysis	Analysis	Outcome	Ref.
Two 5 mm diameter calvarial defects	Sprague–Dawley rats (male)	Blank group: left side defect Hydrogel group (*n* = 12) Hydrogel + Exo (NC) (*n* = 12) Hydrogel + Exo (miR-375) (*n* = 12)	20 μL Exo (miR-375)or Exo (NC) at 50 μg/mL	Hydrogel (250 μL)	8 weeks	μCT, histology, and IHC	↑ BV/TV ratio and BMD; new bone formation; mature osteoid, OCN, and BMP2 by Exo (miR-375).	[38]
Two 5 mm diameter calvarial defects	Sprague–Dawley rats (male)	HA group (*n* = 10) HA + MSC-Exos (*n* = 10) HA + DMOG-MSC-Exos (*n* = 10)	100 μg of exosomes in 200 μL PBS or 200 μL PBS alone	Classical porous hydroxyapatite scaffolds	8 weeks	μCT, histology, sequential fluorescent labeling, and immunofluorescence staining	↑ BV/TV ratio and BMD, new bone area by DMOG-MSC-Exos. ↑ new vessel area and IHC for CD31 by DMOG-MSC-Exos.	[20]
Two 5 mm diameter calvarial defects	Rats (strain/sex NR)	Control group (collagen alone) Positive control group (rhBMP2 + collagen) Control EV group BMP2 EV group (*n* = 6 defects per group and time point)	5 × 10^8^ EVs/50 μL per defect 50 μg/50 μL rhBMP2 per defect	Collagen tape	4, 8, and 12 weeks	μCT, histology, and IHC	↑ BV/TV ratio; ongoing woven bone formation; and early expression of BMP2, BSP, DMP1, and OCN by BMP2 EV. Fatty marrow was not present in BMP2 EV group as in rhBMP2 group.	[21]
Two 5 mm diameter calvarial defects	Sprague–Dawley rats (sex NR)	β-TCP (*n* = 13) BMSC-Exos + β-TCP (*n* = 13) BMSC-Exos-HIF1a + β-TCP (*n* = 13)	200 μg of exosomes	β-TCP	12 weeks	μCT, sequential fluorescent labeling, histomorphology, and IHC	↑ BMD and BV/TV analysis, new bone area, vessel number and volume, and OCN and CD31 expression by BMSC-Exos-HIF1a + β-TCP group.	[18]
Femoral fracture model with Kirschner’s wire	Mice (strain/sex NR)	PBS group (*n* = 8) Exos group (*n* = 8) Hypo-Exos group (*n* = 8)	200 μg of exosomes in 200 μL of PBS or 200 μL PBS alone	PBS	7 days	X-ray, μCT, histology, and immunofluorescence staining	↑ in callus volume/tissue volume, vessel number, vessel volume, and Ki67/CD31-positive cells by Hypo-Exos.	[17]
Segmental radius defect (8 mm)	Sprague–Dawley rats (male)	Healthy group Negative group Exo-D0 group Exo-D4 group Exo-D10 group Exo-D15 group hMSC cell-seeded group (*n* = 5/group)	NR	Poly-L-lysine-coated 3D titanium scaffolds	4 and 12 weeks	Histology	↑ new bone formation, osteoblasts, and Haversian canal-like structures by Exo-10 and Exo-15. ↑ collagen by Exo-15.	[40]
Alveolar bone defects (4 mm length × 3 mm width × 2 mm height)	Sprague–Dawley rats (male)	Matrigel™ group Exo+ Matrigel™ group SM-Exo + Matrigel™ group Control (PBS) group (*n* = 8)	100 μg/100 μL of Matrigel™ or 100 μL of Matrigel™ alone	Matrigel™	3 and 6 weeks	μCT, histology, and IHC	↑ BV/TV ratio, new bone area, and RUNX-2 and OCN expression (IHC) in the SM-Exo + Matrigel group.	[39]
Distal femur defects (3.5 mm diameter × 4 mm depth)	Sprague–Dawley rats (male)	SF-PBS group SF-Exo group SF-β-TCP-Exo group SF-CS-Exo group SF-Sr-CS-Exo group (*n* = 30 femur defects in 15 rats)	100 μL (1000 μg/mL)	Silk fibroin	8 weeks	μCT, histology, and IHC	↑ BV/TV and BMD values; new bone area; and expression levels of CD31, VEGF, and VE-ca in the SF-Sr-CS-Exo group.	[41]

ALP, alkaline phosphatase; ARS, alizarin red staining; BMSCs, bone marrow mesenchymal stem cells; BMD, bone mineral density; BMP, bone morphogenetic protein; BSP, bone sialoprotein; BV/TV, bone volume/total bone volume; DMP1, dentin matrix acidic phosphoprotein 1; DMOG, dimethyloxalylglycine; EVs, extracellular vesicles; Exo, exosome; FEEs, functionally engineered Evs; HA, hydroxyapatite; IHC, immunohistochemistry; MSCs, mesenchymal stem cells; NC, negative control; NR, not reported; OCN, osteocalcin; OM, osteogenic medium; PBS, phosphate-buffered saline; PM, normal growth medium; rhBMP2, recombinant human bone morphogenetic protein; SF, silk fibroin, SM, strain microenvironment; Sr-CS, strontium-substituted calcium silicate; VE-cad, vascular endothelial cadherin; VEGF, vascular endothelial growth factor; β-TCP, tricalcium phosphate; μCT, micro-computed tomography; ↑, upregulated; ↓, downregulated).

**Table 5:** Secondary outcomes related to microRNA/circRNA profile and signaling pathway/gene expression analysis.

microRNA/circRNA Profile	Functions/Signaling Pathways	Reference
Exo-D3 versus Exo-P6: one miRNA differentially expressed, which enriches three pathways related to osteogenic differentiation. Exo-D21 versus Exo-P6: nine differentially expressed miRNAs that enrich twenty pathways related to osteogenic differentiation. Exo-D21 versus Exo-D3: sixteen differentially expressed miRNAs that enrich twenty pathways related to osteogenic differentiation.	Exo-D3 versus Exo-P6: Hippo signaling pathway, adherens junction, ECM–receptor interaction. Exo-D21 versus Exo-P6: Wnt, Hippo, MAPK, cAMP, PI3K-Akt, TGF-beta, TNF, VEGF, insulin, and AMPK signaling pathways, among others. Exo-D21 versus Exo-D3: Wnt, Hippo, MAPK, cAMP, PI3K-Akt, TGF-β, TNF, HIF-1, insulin, and AMPK signaling pathways, among others.	[36]
11 circRNAs were upregulated in Exo7. ↑ circLPAR1 and ↓ hsa-miR-31 in Exo7-treated DPSCs	circLPAR1 was predicted to bind to hsa-miR-31, a miRNA that showed an inhibitory effect against osteogenic differentiation. circLPAR1 would be the target of hsa-miR-31. Both downregulation of hsa-miR-31 and upregulation of circLPAR1 promoted osteogenic differentiation of DPSCs.	[31]
↑ of 72 miRNAs and ↓ of 35 miRNAs in exosomes derived from osteogenically differentiated PDLSCs. ↑of miR-122-5p, miR-142-5p, miR-25-3p, miR-192-5p. ↓ of miR-125b-5p, let-7b-5p, and miR-100-5p.	Predicted functions: catalytic activity, protein binding, metabolic process, transport, and phosphate-containing compound metabolic process. Processes related to target genes: 2-oxocarboxylic acid metabolism, adipocytokine signaling pathway, AMPK signaling pathway, insulin signaling pathway, and MAPK signaling pathway.	[33]
Upregulation of 160, 166, 193, and 136 miRNAs and downregulation of 130, 139, 150, and 191 miRNAs were were observed in the Exo-D4, Exo-D10, Exo-D15, and Exo-D20 exosomes, respectively. ↑ expression of osteogenic miRNAs (Hsa-miR-146a-5p, Hsa-miR-503-5p, Hsa-miR-483-3p, and Hsa-miR-129-5p) and ↓ expression of anti-osteogenic miRNAs (Hsa-miR-32-5p, Hsa-miR-133a-3p, and Hsa-miR-204-5p) in 10- and 15-day osteogenically induced exosomes.	Predicted signaling pathways: PI3K/Akt and MAPK.	[40]
↑ of 201 miRNAs and ↓ of 33 miRNAs in exosomes derived from osteogenically differentiated ASCs. ↑ of five miRNAs (miR-130a-3p, miR-30b-5p, miR-34a-5p, miR-324-5p, and miR-378f) and ↓ of miR-513b-5p.	Predicted processes affected: axon guidance, MAPK signaling, Wnt signaling, endocytosis, regulation of actin cytoskeleton, and TGF-β signaling pathway. Functions affected: enzyme binding, cell projection, transcription factor activity, regulation of gene expression, and cell metabolism. Mir-130a03p had the highest differential expression and was predicted to bind to SIRT7. The downregulation of SIRT7 may enhance the osteogenic differentiation of BMSCs.	[34]
NA	Phosphorylation of STAT6, GSK-3α/β, STAT5b, and STAT5a/b increased following stimulation with 0d-Exo, whereas phosphorylation levels of FAK, PRAS40, and WNK1 were downregulated. SMAD 4 and BMPR2 were upregulated in 0d-Exo treatment. SMAD1/8, BMPR1A, and BMPRIB showed no difference between treatments.	[35]
D21 versus D0 (do not specify the type of cell): ↑ of miR-186, miR-210, miR-181c-5p, and miR-146a-5p and ↓ of miR-133 and miR-485.	Potential signaling pathways: TGF-beta signaling pathway, Hippo signaling pathway, Map kinase, and Wnt signaling pathway, among others.	[32]
NA	↓ PTEN in DMOG-MSC-Exos. The deficiency of PTEN is related to increased migration and invasion of HUVECs. The downstream target of PTEN, AKT/mTOR, was blocked, and DMOG-MSC-Exos lost their superior pro-angiogenic abilities.	[20]
↑ of miR-146a in BMSCs by Sr-CS extracts	The miR-146a inhibition led to the downregulation of miR-146a in both BMSCs and BMSC-Exos. Treatment with Sr-CS + 146I-Exo (derived from miR-146a inhibition) diminished its angiogenic ability in HUVECs. Prediction targets showed that miR-146a directly targets Smad4 and NF2 (reported to inhibit angiogenesis).	[41]
	↓ of IGFBP3 associated with miR-375 overexpression. ↑ cell differentiation in IGFBP3-deficient cells cultured in OM. Furthermore, the increase in osteogenic differentiation induced by Exo (miR-375) treatment was reversed by IGFBP3 recombinant treatment.	[38]
NA	BMP2 FEEs were able to trigger SMAD 1/5/8 phosphorylation, and control EVs had no effect beyond the resting cell state.	[21]
↑ of 94 miRNAs and ↓ of 39 miRNAs in the Hypo-Exos group when compared to the Exos group. ↑ of miR-126, miR-855–5p, miR-146b, miR-223, and miR-451.	Knockdown of miR-126 inhibited Hypo-Exos-mediated proliferation, migration, and angiogenesis in vitro and in vivo. SPRED1 is a target of miR-126 and was increased in miR-126 knockdown Hypo-Exos-treated cells. Silencing of SPRED1 promoted cell proliferation, migration, and tube formation during treatments with miR-126 knockdown Hypo-Exos. Hypo-Exos suppressed SPRED1 by activating the Ras/Erk pathway.	[17]
565 miRNAs were differentially expressed in SM-Exo. ↑ of 16 miRNAs and ↓ of 9 miRNAs in SM-Exo compared to Exos. ↑ of mir-10a-5p and mir-10b-5p and ↓ of mir-212-3p in SM-Exo.	NA	[39]

ALP, alkaline phosphatase; AMPK, AMP-activated protein kinase; ARS, alizarin red staining; ASCs, adipose stem cells; BMSCs, bone marrow mesenchymal stem cells; BMP2, bone morphogenetic protein 2; BMPR2, bone morphogenetic protein receptor type 2; circRNA, circular RNA; DMOG, dimethyloxalylglycine; ECM, extracellular matrix; EVH1 domain-containing protein 1; Exo, exosome; FAK, focal adhesion kinase; FEEs, functionally engineered extracellular vesicles; GSK, glycogen synthase kinase; HUVECs, human umbilical vein endothelial cells; IGFBP3, insulin-like growth-factor-binding protein 3; IRS1, insulin receptor substrate 1; JAK-STAT, Janus kinase (JAK) signal transducer and activator of transcription; LPAR1, lysophosphatidic acid receptor 1; miRNA, microRNA; MAPK, mitogen-activated protein kinase; NA, not applicable; NF2, neurofibromatosis type 2; OM, osteogenic medium; PDLSCs, periodontal ligament stem cells; PDSCs, dental pulp stem cells; PLK2, Polo-like kinase 2; PRAS40, proline-rich AKT substrate of 40 kDa; PTEN, phosphatase and tensin homolog; SIRT7, sirtuin 7; SM-Exo, strain-microenvironment-derived exosome; SPRED1, Sprouty-related; Sr-CS, strontium-substituted calcium silicate; STAT, signal transducer and activator of transcription; TGF-β, transforming growth factor beta; TNF, tumor necrosis factor; VEGF, vascular endothelial growth factor; WNK1, lysine deficient protein kinase 1; ↑, upregulated; ↓, downregulated).

## Data Availability

Not applicable.

## Supplementary Materials

The following supporting information can be downloaded at: https://www.mdpi.com/article/10.3390/biology11050733/s1, Table S1: Study characteristics.

## Appendix A

Electronic search strategy
Pubmed:
#1: (exosomes[Title/Abstract] OR “extracellular vesicles”[Title/Abstract]) OR (EVs[Title/Abstract])) OR (microvesicles[Title/Abstract])
#2: (“mesenchymal stem cells”[Title/Abstract]) OR (“mesenchymal stromal cells”[Title/Abstract])) OR (“stem cells”[Title/Abstract])) OR (MSCs[Title/Abstract])))
#3: (“Bone regeneration”[Title/Abstract]) OR (“Bone healing”[Title/Abstract])) OR (“Bone repair”[Title/Abstract])) OR (osteogenesis[Title/Abstract])) OR (fracture[Title/Abstract])
#4: #1 AND #2 AND #3
Scopus:
#1: TITLE-ABS-KEY (exosomes OR “extracellular vesicles” OR EVs OR microvesicles)
#2: TITLE-ABS-KEY (osteogenesis OR “bone regeneration” OR fracture OR “bone healing” OR “bone repair”)
#3: TITLE-ABS-KEY (“mesenchymal stem cells” OR “mesenchymal stromal cells” OR “stem cells” OR mscs))
#4: #1 AND #2 AND #3 (LIMIT-TO (DOCTYPE, “ar”)) AND (LIMIT-TO (LANGUAGE, “English”))
Web of Science:
#1: TS = (exosomes OR “extracellular vesicles” OR EV OR microvesicles)
#2: TS = (osteogenesis OR “bone regeneration” OR fracture OR “bone healing” OR “bone repair”)
#3: TS = (“mesenchymal stem cells” OR “mesenchymal stromal cells” OR “stem cells” OR MSCs)
#4: #1 AND #2 AND #3 (Refined by: DOCUMENT TYPE: (ARTICLE OR EARLY ACCESS).
