# Supplementary material for: Preconditioning Methods to Improve Mesenchymal Stromal Cell-Derived Extracellular Vesicles in Bone Regeneration—A Systematic Review
Source: Biology (Basel). 2022 May 11;11(5):733. doi: 10.3390/biology11050733 (PMC9138769; doi:10.3390/biology11050733)
Supplement: Supplementary file 1 [file biology-11-00733-s001.zip › Systematic review Protocol - StArt.pdf]

StArt - State of the Art through Systematic Review

Approaches to improve the therapeutic effects of mesenchymal stromal cell-derived extracellular vesicles in bone regeneration – a systematic review

Date: 12/20/2021 - 12:59:27

**Title:**	Approaches to improve the therapeutic effects of mesenchymal stromal cell-derived extracellular vesicles in bone regeneration – a systematic review
**Researchers:**	Fernanda Campos Hertel
**Objectives:**	Ponctuate and assess the methodology used to improve the therapeutic abilyties of mesenchymal stromal cells derived extracellular vesicles
**Main Question:**	Does the conditioning of MSCs improve the therapeutic effects of MSC-derived EVs for bone regeneration?
**Intervention:**	Treatment with EVs derived from preconditioned MSCs
**Control:**	Treatment withl EVs without conditioning
**Population:**	in vivo or in vitro studies with a focus on osteogenesis/bone regeneration
**Results:**	osteogenesis/angiogenesis for in vitro and in vivo studies
**Keywords:**	MSC; bone healing; bone regeneration; bone repair; exosome; extracellular vesicles; fracture; mesenchymal stem cells; mesenchymal stromal cells
**Source Selection Criteria:**	specific database; broad-ranging database;
**Studies Languages:**	English;
**Source Search Methods:**	Included in the manuscript apendix
**Source Engine:**	PubMed; Web of Science; Scopus;
**Studies inclusion and exclusion criterias:**	(E) articles that reported the use of EVs from other cells sources than MSCs; (E) articles that reported the use of an animal model with induced disease (as osteoporosis, obesity, femoral head necrosis, periodontal related diseases); (E) articles that did not use EVs as treatment; (E) treatment associating MSCs with EVs; (E) articles that did not report a control group of EVs; (E) articles that did not report conditioning of MSCs; (E) review articles, conference abstracts;<br>(I) articles that reported the treatment with pre-conditioned MSC-derived EVs in vitro and/or in vivo;<br>(I) ; articles that reported the assessment of osteogenesis in vitro and/or in vivo;
**Studies types definition:**	All types, if the inclusion criteria was met.;
**Initial studies selection:**	Based on title and abstract;
**Information Extraction Fields:**	Journal; Country; year of publication;<br>Type of MSC; Species of MSC;<br>Type of vesicle=[exosome,extracellular vesicle,microvesicles];<br>Type of conditioning method?; Scaffolf? ={yes,no}; Type of scaffold;<br>Isolation method; Characterization methods; Type of study=[in vitro,in vivo]; In vitro: celular type for in vitro analysis; In vitro assays; Animal model; in vivo: type of defect; treatment groups for in vivo analysis;<br>Extracellular vesicles concentration; vehicle for in vivo application; in vivo delivery route;<br>application frequency; time of euthanasia; Types of in vivo analysis; in vitro outcome: uptake; in vitro outcome: proliferation assay; in vitro outcome: osteogenic differentiation; in vitro outcome: scratch wound assay; in vitro outcome: osteogenic markers; in vitro outcome: miRNAs exosomal; in vivo outcome: microct; in vivo outcome: x-ray; in vivo outcome: histology; in vivo outcome: IHC; in vivo outcome: immunofluorescence;
**Results Summarization:**	Descriptive, tables and figures;
