# Supplementary material for: Preconditioning Methods to Improve Mesenchymal Stromal Cell-Derived Extracellular Vesicles in Bone Regeneration—A Systematic Review
Source: Biology (Basel). 2022 May 11;11(5):733. doi: 10.3390/biology11050733 (PMC9138769; doi:10.3390/biology11050733)
Supplement: Supplementary file 1 [file biology-11-00733-s001.zip › Table S1.pdf]

Table S1. Studies identification.

Year	Title	Journal	Country	Author
**2016**	Hijacking the cellular mail: exosome mediated differentiation of mesenchymal stem cells	Stem Cells International	USA	[37]
**2017**	Exosomes secreted from mutant-HIF-1α-modified bone-marrow-derived mesenchymal stem cells attenuate early steroid-induced avascular necrosis of femoral head in rabbit	Cell Biology International	China	[28]
**2018**	Tissue-engineered bone immobilized with human adipose stem cells-derived exosomes promotes bone regeneration	ACS Applied Materials & Interfaces	China/The Netherlands	[29]
**2018**	Mesenchymal stem cell-derived exosomes have altered microRNA profiles and induce osteogenic differentiation depending on the stage of differentiation	Plos One	Sweden	[36]
**2018**	Dexamethasone-Activated MSCs Release MVs for Stimulating Osteogenic Response	Stem Cells International	China	[30]
**2019**	Exosomes derived from miR-375-overexpressing human adipose mesenchymal stem cells promote bone regeneration	Cell Proliferation	China	[38]
**2019**	Dimethyloxaloylglycine-stimulated human bone marrow mesenchymal stem cell-derived exosomes enhance bone regeneration through angiogenesis by targeting the AKT/mTOR pathway	Stem Cell Research and Therapy	China	[20]
**2019**	Immunoregulatory role of exosomes derived from differentiating mesenchymal stromal cells on inflammation and osteogenesis	Journal of Tissue Engineering and Regenerative Medicine	Australia/ China	[35]
**2019**	Promoting osteogenic differentiation of human adipose-derived stem cells by altering the expression of exosomal miRNA	Stem Cells International	China	[34]
**2020**	Functionally engineered extracellular vesicles improve bone regeneration	Acta Biomaterialia	USA	[21]
**2020**	Human periodontal ligament stem cell-derived exosomes promote bone regeneration by altering microRNA profiles	Stem Cells International	China	[33]

**2020**	Hypoxic mesenchymal stem cell-derived exosomes promote bone fracture healing by the transfer of miR-126	Acta Biomaterialia	China	[17]
**2020**	Comparison study of stem cell-derived extracellular vesicles for enhanced osteogenic differentiation	Tissue Engineering: Part A	USA/Iran	[32]
**2020**	Exosomal circlPAR1 promoted osteogenic differentiation of homotypic dental pulp stem cells by competitively binding to hsa-miR-31	BioMed Research International	China/ Thailand	[31]
**2020**	BMSC-exosomes carry mutant HIF-1a for improving angiogenesis and osteogenesis in critical-sized calvarial defects	Frontiers in Bioengineering and Biotechnology	China	[18]
**2020**	Three-dimensional mechanical microenvironment enhanced osteogenic activity of mesenchymal stem cells-derived exosomes	Chemical Engineering Journal	China	[39]
**2020**	Human mesenchymal stem cell-derived exosomes enhance cell-free bone regeneration by altering their miRNAs profiles	Advanced Science	USA/China	[40]
**2021**	Bone marrow stromal cells stimulated by strontium-substituted calcium silicate ceramics: release of exosomal miR-146a regulates osteogenesis and angiogenesis	Acta Biomaterialia	China	[41]

USA, United States of America; HIF, hypoxia-inducible factor; MSCs, mesenchymal stem cells; miRNA, microRNA; BMSC, bone marrow mesenchymal stem cells.
